# Description of a myxozoan parasite *Myxobolus* sp.n PKB2014 from an edible fish, with emphasis on its molecular characterization

**Published:** 2015-12

**Authors:** Somerita Panda, Subarna Ghosh, Probir Kumar Bandyopadhyay

**Affiliations:** Parasitology Laboratory, Department of Zoology, University of Kalyani, Kalyani-741235 West Bengal, India

**Keywords:** *Myxobolus* sp.n PKB2014, West Bengal, Labeo rohita, 18S rRNA, Phylogenetic relationship

## Abstract

In India, more than 104 species of *Myxobolus* have so far been reported infecting freshwater as well as marine fishes. The study focuses on the description of a new myxosporean species, *Myxobolus* sp.n PKB 2014 from the gill lamellae of an Indian major carp *Labeo rohita.* The species have been described on the basis of morphological characterization of the spores, tissue architecture and 18S rDNA sequence data. The plasmodia of *Myxobolus* sp.n PKB 2014 were round in shape measuring, 50 to 70 μm in diameter and spores were ellipsoidal in frontal view measures 14.7 ± 0.51 μm. The 18S rRNA nucleotide sequence with 806 bp of *Myxobolus* sp.n PKB 2014 (Accession number KJ652226) clustered phylogenitically with other *Myxobolus* spp. infecting cyprinid gills with 90-99% similarity. According to the phylogenetic study we concluded that *M. wulli* was the closest relative having 99% similarity with the species under description but the sequence was distinct in each species which additionally exhibited different morphological features. The infection rate was low to moderate. After through comparison it can be concluded that the species being described here is new to science which is designated as *Myxobolus* sp.n. PKB 2014.

## INTRODUCTION

Myxozoans are economically important group of microscopic metazoan parasites, which cause disease in a large variety of commercially important fishes. They have also been reported in platyhelminthes, reptiles, amphibians, mammals as well as in faecal sample of human beings [1]. Nowadays research on myxozoan fish parasites gets momentum in the field of ichthyoparasitology. Many myxosporeans, which have been reported from freshwater as well as marine fishes, are highly pathogenic. Among myxosporeans, the genus *Myxobolus *includes the highest number of species. Eiras et al. reported 751 valid species, while Lom and Dyková counted 792 known species [2, 3]. The genus *Myxobolus *was first established by Bütschli [4] having spores with or without an iodinophilous vacuole and with one or two polar capsules. The myxozoan spores having two polar capsules, with or without iodinophilus vacuoles and generally two sporogenic nuclei have been observed and placed under the Genus *Myxobolus *Bütschli [4]. Kalavati reported more than 104 species of *Myxobolus* sp. from freshwater as well as marine fishes from India [5]. Most of the myxosporean parasites have been found in the tropical and subtropical countries [6-17]. Recently, Kaur and Singh described fifteen new species of the genus *Myxobolus* from freshwater fishes of the Wetlands of Punjab [18-27]. The present study focuses on the description of one new species belonging to the genus *Myxobolus *Bütschli [4] isolated from the gills of *Labeo rohita *(Ham.) collected from fish farms of South 24 Parganas, Kolkata, West Bengal, India. The description of the species has been made according to Lom and Arthur [28].

A good number of *Myxobolus* have so far been described earlier from the same host fish, *Labeo rohita* (Hamilton) collected from the fish farms of South 24 Parganas (22.4200°N, longitude:88.4200°E), West Bengal, but little work has been done on rRNA genes. Although identification on the basis of phenotypic characteristics is inconclusive, molecular identification along with phylogenic description is more precise method for characterising new species. In the present study, integrated approaches including phenotypic and molecular analysis of the 18S rRNA region of LSU (Large SubUnit: 18S rDNA) were utilised to isolate the *Myxobolus* species from fresh water fish, *Labeo rohita* of West Bengal, India. Histopathology and electron microscopic studies of the affected organs of the fish were performed to observe the nature of damage caused by the parasite.

## MATERIALS AND METHODS

**Fish sampling:** *Labeo rohita* were collected from different fish farms of South 24 Parganas districts (22.4200° N, longitude: 88.4200°E) of West Bengal, India, during the tenure of the research work i.e. June-August 2013; and were transferred alive to the laboratory for detection of myxozoan infection. The external body surface of the fish was examined for the infection. Many grossly visible whitish spots were detected in the gill lamellae. Detailed (microscopic) investigations were performed on the gills. The plasmodia were carefully removed from the gill lamellae.

**Sample preparation and Morphometric Analysis: **The cysts attached to the gill lamellae of *Labeo rohita* were isolated. Some of the cysts were taken on glass slides and slightly ruptured on one end with a sharp needle. The spores were released from cyst smeared on slides with few drops of distilled water, covered with cover slips and sealed with DPX for examination of fresh spores under oil immersion lens of Motic BA400 microscope.Other spores obtained from the same cyst were fixed in 70% ethanol in vials for further morphological and molecular biological examination. Permanent mounting of myxosporen parasites were done by staining with Giemsa. Air dried smears were treated with absolute methyl alcohol for about 8 min to fix the parasites and dried again. The stock solution of Giemsa was diluted with water in the ratio of 1: 2. The slides were covered with dilute Giemsa stain for 40 minutes. The slides were finally washed by pouring neutral distilled water (until no noticeable extent colour was detected). The slides were then air dried. The slides containing myxosporean spores were observed under the Motic BA400 microscope with inbuilt digital camera. 

**Light** **microscopy: **Infected gill lamellae were fixed in Bouin’s solution for 4 hours, washed in 80% ethanol several times, embedded in paraffin wax, cut into 5 to 8 μm thick sections and stained with hematoxylin and eosin. Photomicrographs of histological sections were taken using olympus microscope (Olympus CX41) equipped with a digital camera. Measurements of fresh spores were taken following the guidelines of Lom and Arthur [28].

**Scanning electron microscopy: **To conduct SEM study both the cyst and the infected gill lamellae were processed and then fixed in 2.5% glutaraldehyde solution for two hours at 4 °C followed by dehydration with ethanol and washing with absolute acetone and amyl acetate mixture in 3:1, 2:2 and 1:3 ratios, respectively. The final wash was done in 100% amyl acetate. The gill tissues and cyst were then processed with HCP: 2 Critical Point Dryer (Hitachi) using CO_2_. The tissues were coated with metallic gold in an IB-2 ion coater and examined in a Hitachi S-530 scanning electron microscope at accelerating voltages of 15 and 20KV.

**Molecular characterization and Phylogenetic analysis: **The plasmodia filled with mature spores were ruptured by a sharp needle and the contents were collected carefully .The parasite DNA was extracted and then ruptured by a sharp needle and the contents were collected. The spores were then centrifuged at 1000×g for 10 min. The DNA of the parasite was extracted by suspending the spores in 500 µl lysis buffer (100 mM NaCl, 10 mM Tris, 10 mM EDTA, 0.2% SDS, 0.4 mg/ml Proteinase K) and incubating overnight at 55°C. Then, 500 µl of phenol: chloroform (1:1) was added to the digested spores, mixed gently, and centrifuged at 5200×g for 10 min. The extraction step was repeated followed by phenol: chloroform (1:1) treatment. The DNA was precipitated at –20°C throughout the night and pelleted by centrifugation at 10000×g for 30 min. The pellet was washed once with 70% ethanol, air-dried for several minutes and re-suspended in molecular biology grade water. 

The 18S small subunit ribosomal RNA (18S rRNA) was amplified by Gradient PCR system (Eppendorf Master cycler Pro S) using a set of universal eukaryotic primers - UEP-F, 5´-ACC TGG TTG ATC CTG CCA G-3´ and UEP-R, 5´-CTT CCG CAG GTT CAC CTA CGG-3´ [29]. The PCR was run using a mixture containing 50 ng of genomic DNA, 10 μM of each primer, 2x PCR TaqMixture (HiMedia, Mumbai). Amplification was done by initial denaturation at 95°C for 5 min, followed by 35 cycles of denaturation at 95°C for 30 sec, annealing of primers at 51°C for 30 sec and extension at 72°C for 60 sec. The final extension was at 72°C for 5 min. The PCR products were analysed on a 1.5% agarose (HiMedia, Mumbai) gel containing 0.5 μg/ml ethidium bromide in 1×Tris-acetate- EDTA (TAE) buffer. Purified PCR products were sequenced in both directions. The PCR amplified products were sequenced at the Genomics Division, Xcelris Labs Ltd, Ahmadabad, India. The amplified PCR product was first purified using EXO-SAP treatment. The concentration of the purified DNA was determined and subjected to automated DNA sequencing on ABI 3730xl Genetic Analyzer (Applied Biosystems, USA). Sequencing was carried out using BigDye® Terminator v3.1 Cycle sequencing kit (Applied Biosystems, USA) following manufacturers’ instructions. Electrophoresis and data analysis were carried out on the ABI 3730xl Genetic Analyzer. Phylogenetic analyses were performed on a selection of ten 18S rRNA gene sequences, including the novel sequence, closely related sequences determined by Basic Local Alignment Search Tool (BLAST). The nucleotide sequence generated in the present study has been deposited in the GenBank database under accession number KJ652226. Multiple alignments and data analysis were performed by ClustalX [30] and MEGA5 software [31], respectively. Genetic distance analyses were conducted using the Kimura 2-parameter model [32]. Codon positions included were 1st+2nd+3rd+Noncoding. All positions containing gaps and missing data were eliminated. The evolutionary distances were computed using the Kimura 2-parameter method [32] and were in the units of the number of base substitutions per site. There were a total of 792 positions in the final dataset. Evolutionary analyses were conducted in MEGA5 [31]. The alignment was corrected manually using the alignment editor. The accession numbers of the sequences analyzed in this study are given in the figures showing phylogenetic tree .Regions judged to be poorly aligned and characters with gap in any sequences were excluded from subsequent analysis for 18S rDNA. The evolutionary history was inferred using the bootstrap method [33]. The bootstrap consensus tree inferred from 500 replicates have been taken to represent the evolutionary history of the taxa analysed [34]. Branches corresponding to partitions reproduced in less than 50% bootstrap replicates had been collapsed. The percentage of replicate trees in which the associated taxa clustered together in the bootstrap test (500 replicates) is shown next to the branches.

## RESULTS AND DISCUSSION

**Figure 1:**
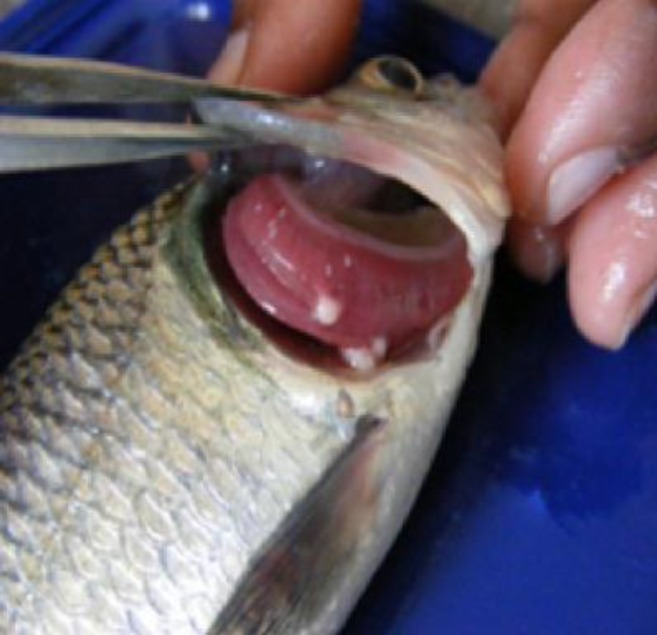
Myxosporidian infections in fish are shown

The cysts of* Myxobolus* were found attached with the gill lamellae of the *Labeo rohita *(Fig. 1). Cysts were creamy white in colour and round in shape. They contained both late developmental stages and mature spores

**Figure 2:**
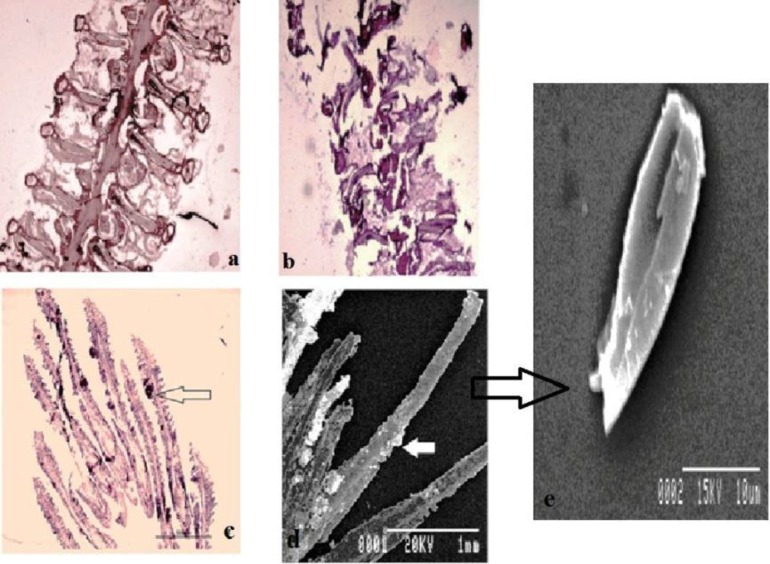
Photograph showing the histological section of gill lamellae of host fish *Labeo rohita* (HE-stained); a) Gill lamella of healthy *Labeo rohita*; b) histological sections cysts of *Myxobolus* sp.n PKB2014 attached to the gill lamellae. Gill lamella of healthy *Labeo rohita*, photograph showing the deformed gill lamellae of host fish (*Labeo rohita*) by attachment of the parasite; c) Round-shaped plasmodia attached to gill intra-lamellar sections of the host; d) scanning electron microscopic photomicrographs of plasmodia attached to the gill lamellar of the host fish; e. Ultra-structural study of *Myxobolus* sp.n PKB2014 spore

Scanning electron microscopic study along with histological examination revealed aggregation of cysts attached to the gill lamellae. Round-shaped plasmodia of 50 to 70 μm diameter (Fig. 2e) were found intra-lamellar in the gills (Fig. 2c and d). Mature cysts deformed the neighbouring lamellae by compressing them (Fig. 2b). Myxozoan infection was found in 5 out of the 20 fish samples examined. Plasmodia with mature *Myxobolus* sp.nPKB2014 spores under study were found in the gill lamellae of *Labeo rohita.*

**Figure 3:**
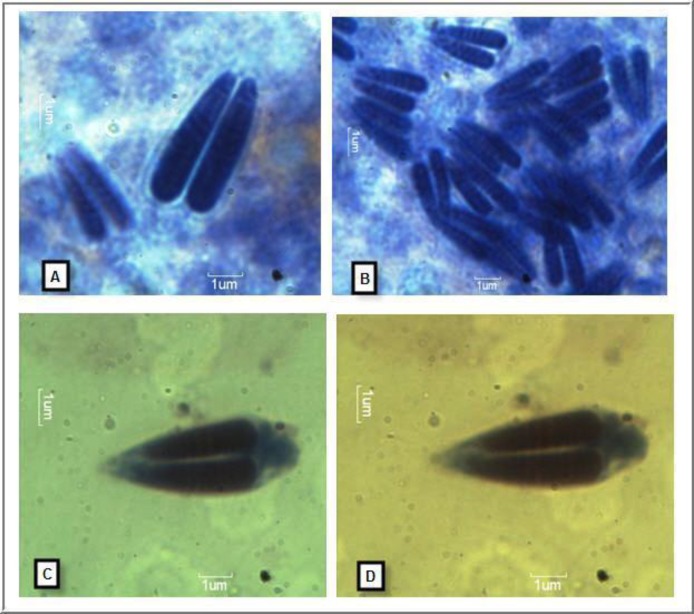
(A-D) Light microscopic Photomicrographs of *Myxobolus*.sp.n PKB2014 stained with Giemsa (unextruded) (Scale bar: 1µm

The shapes of the spores were ellipsoidal, while observed it from frontal side. The size of spores was measured. Regarding length they were in the range of 11.1 to 19.1 μm (mean: 14.7 ± 0.51, n=20) and regarding width they were in the range of 5.3 to 7.3 μm (mean: 6.36 ± 0.53, n=20) (Fig. 3 and Table 1).

Polar capsules were elongated, different in size, slightly converging anteriorly. Regarding the length of the larger polar capsule, it ranges from 8.2 to 14.2 μm (mean: 10.64 ± 0.40, n = 20) and regarding its width it measures 2.99 ± 0.14 μm (range, 2.3 to 3.9, n=20) width. Smaller capsule were 9.55 ± 0.42 μm (range 7.1 to 12.9, n=20) length and 2.79 ± 0.13 μm (range, 2.2 to 3.6) width (Table.1). The spore index of LS: WS = 1: 0.4326; LPC: WPC = 1:0.281; LSPC: WSPC = 0.2921. Polar filament coils were invisible in the capsules. No inter-capsular appendix has been observed.

**Table 1:** Measurements of *Myxobolus* sp.n. PKB2014 spores isolated from gills of *Labeo rohita*

**Parameters **	**Range **	**Mean **	**Standard Deviation (SD)**
**Length of the spore (LS) **	11.1 -19.1	14.7	0.51
**width of the spore (WS) **	5.61-6.12	6.36	0.53
**Length of Larger Polar Capsule (LLPC) **	8.2 -14.2	10.64	0.40
**width of Larger Polar Capsule (WLPC) **	2.3 - 3.9	2.99	0.14
**Length of Smaller Polar Capsule (LSPC) **	7.1- 12.9	9.55	0.42
**width of Smaller Polar Capsule (WSPC) **	2.2 - 3.6	2.79	0.13

**Figure 4:**
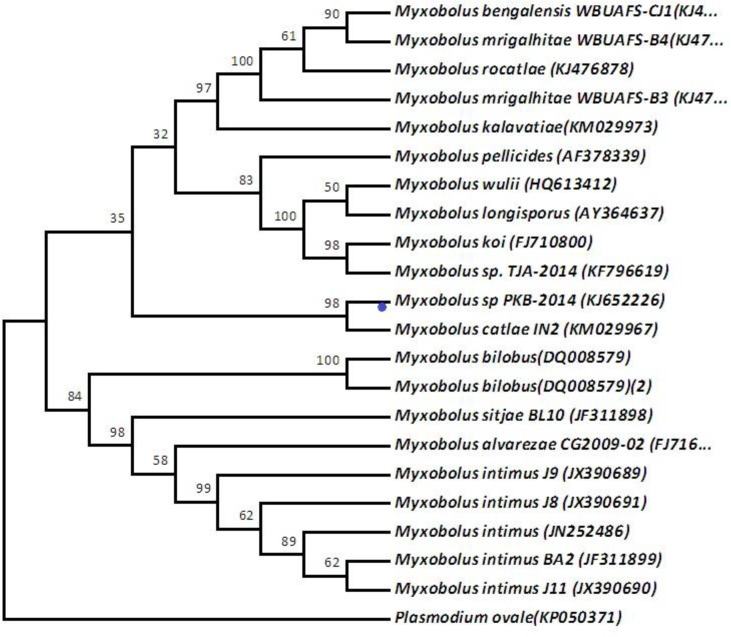
Phylogenetic tree based on the 18S rRNA sequence

**Molecular comparison: **The universal primer sets UEP-F and UEP-R were used to amplify 806 bp of the 18S rRNA gene from *Myxobolus* sp. n. PKB2014.It has been found from the phylogenetic tree (Fig. 4) that the 18S rRNA of *Myxobolus* sp. n. PKB2014 was related to another gill-infecting species described from *Labeo rohita*. Based on the nucleotide sequences, *M. wulli* (GenBank accession no. HQ613412.1) shares 99% similarity with *Myxobolus* sp. n. PKB2014. A 97% similarity with *M. toyamai* (infecting the gills of *Cyprinus carpio*), 96% similarity with *M. koi* (FJ710800.1), *M. pyramidis* (HQ613411.1), *M. longisporus* (AY364637.1), 94% similarity with *M. ampullicapsulatus* and 91% similarity with *M. pellicides* have been observed. The similarity with *M. bilobus* (DQOO8579) was described from Ontorio, Canada, while the similarity with *M. catlae *(KM029967) and *Myxobolus ampullicapsulatus *were reported (KC425225.1) from China, and with the out group such as *Thelohanellus toyamai* (HQ338729.1) has also been recorded in the maximum likelihood tree. *Myxobolus intimus *(JX390691.1), *M. alvarezae *CG2009-02 and *M. sitjae* BL10 were distantly related with *Myxobolus* sp. n. PKB2014.This is the first report on molecular characterization of the *Myxobolus* sp. n. PKB2014 species collected from the gill lamellae of the carp,* Labeo rohita *

The identification of different species of *Myxobolus *is very difficult due to great similarity of the spores. Researches can morphologically determine identical spores based on host specificity and also based on the location of spores in the host; however, it is more authentic to characterize the spores on the basis of molecular data. The *Myxobolus *sp.n.PKB2014 showed similarities with *M. andhrae *[35 -36], *M. indirae* [37-36],* M. mahendrae* [38], *M. vedavatiensis* [39] and *M. calbasui* [17] regarding morphometric data. The study of evolutionary history enables us to understand the evolution of modern species and supports some uncertain topologies. In the current work, the evolutionary history was inferred using the Phylogenetic tree generated by maximum likelihood of 18S rRNA gene sequence of PKB2014. The prevalence of *myxobolus *infection on the gills of *L. rohita *[40] reported here was 25%. The incidence of myxosporean infection on gills was usually accompanied by poor growth, uneven size, big head, and poor body muscle ratio of fish hosts, like *L. rohita *and other Indian major and minor carps inhabiting fish ponds; though major disease symptoms were not recorded. At the same time, high frequency of myxosporean infections could become an economic problem due to decrease of fish commercial value. 

In conclusion, the *Myxobolus *species obtained from the gills of the freshwater fish *Labeo rohita *[40] was supposed to be a novel finding and hence proposed as *Myxobolus sp.n *PKB2014 in this communication on the basis of the morphological differences from its closely related species and 18S rRNA sequencing studies.

## Notes

**Acknowledgement: **The authors (SP and SG) are thankful to the University of Kalyani, Kalyani, West Bengal, India and University Grants Commission, New Delhi respectively for financial support. 

## References

[1] Boreham RE, Hendrick S, O’Donoghue PJ, Stenzel DJ (1998). Incidental finding of Myxobolus spores (Protozoa: Myxozoa) in stool samples from patients with gastrointestinal symptoms. J Clin Microbiol.

[2] Eiras JC, Molnár K, Lu YS (2005). Synopsis of the species of Myxobolus Bütschli, 1882 (Myxozoa: Myxosporea: Myxobolidae). Syst Parasitol.

[3] Lom J, Dyková I (2006). Myxozoan genera: definition and notes on taxonomy, life-cycle terminology and pathogenic species. Folia Parasitol.

[4] Bütschli O (1882). Myxosporidea. Bornn’s Klass Ordn., des Tierreiches, Protozoa.

[5] Kalavati C, Nandi NC (2007). Handbook of Myxosporidean parasites of Indian fishes.

[6] Bandyopadhyay PK, Hemananda T, Mitra AK, Mohilal N (2006/7). Myxobolus dhanachandi sp. n. (Myxozoa, Myxosporea, Bivalvulida) from an Indian freshwater fish Channa orientalis (Bloch- Schneider). Protistology.

[7] Basu S, Haldar DP (2003). Three new species of Myxobolus Butschli, 1882 from different food fishes of West Bengal, India. Acta Protozool.

[8] Basu S, Haldar DP (2004). Description of three new myxosporean species (Myxozoa: Myxosporea: Bivalvulida) of the genera Myxobilatus Davis, 1944 and Myxobolus Butschli, 1882. Acta Protozool.

[9] Sarkar NK (1985). Myxosporidian Henneguya mystusia sp. n. (Myxozoa: Myxosporea) from the gills of a freshwater teleost fish Mystus sp. Acta Protozool.

[10] Sarkar NK (1986). On two new species of Myxobolus Butschli, 1882 (Myxozoa, Myxosporea) from the fresh-water fishes of West-Bengal, India. Acta Protozool.

[11] Sarkar N K (1995). Observations on two new myxosporidia (Myxozoa, Myxosporea) from fishes of Bhery-Fishery of West-Bengal, India. Acta Protozool.

[12] Sarkar NK, Mazumder SK, Pramanik A (1985). Observation of four new species of myxosporidia (Myxozoa) from channid (Ophiocephalid) fishes of West Bengal, India. Arch Protistenkd.

[13] Seenappa D, Manohar L (1981). Five new species of Myxobolus (Myxosporea, Protozoa), parasitic in Cirrhina mrigala (Hamilton) and Labeo rohita (Hamilton), with a note on a new host record for Myxosporea curmucae Seenappa and Manohar. J Protozool.

[14] Lalitha-Kumari PS (1965). On a new species of Henneguya (Protozoa: Myxosporidia) from Indian freshwater fish, Ophiocephalus gachua. Riv Parassitol.

[15] Lalitha-Kumari PS (1969). Studies on parasitic protozoa (Myxosporidia) of freshwater fishes of Andhra Pradesh, India. Riv Parassitol.

[16] Tripathi YR (1952). Studies on parasites of Indian fishes. Protozoa: Myxosporidia together with a check-list of parasitic protozoa described from Indian fishes. Rec Indian Mus.

[17] Chakravarty MM (1939). Studies on Myxosporidia from the fishes of Bengal, with a note on myxosporidian infection in aquaria fishes. Arch Protistenkd.

[18] Kaur H, Singh R (2008). Observations on one new species, of genus Myxobolus - M naini and redescription of M magauddi recorded from freshwater fishes of Kanjali Wetland of Punjab, India. Proc 20th Nat Cong Parasitology.

[19] Kaur H, Singh R (2009). A new myxosporean species, Myxobolus eirasi sp. nov., a known species M. venkateshi Seenappa, Manohar, 1981 from the Indian major carp fish Cirrhinasmrigala (Ham). Protistology.

[20] Kaur H, Singh R (2010). A new myxosporean species Myxobolus sclerii sp. nov., one known species M stomum Ali et al., 2003 from two Indian major carp fishes. J Parasit Dis.

[21] Kaur H, Singh R (2010). One new myxosporidian species, Myxobolus slendrii sp nov, one known species, M punjabensis Gupta, Khera, 1989 infecting freshwater fishes in wetlands of Punjab, India. Parasitol Res.

[22] Kaur H, Singh R (2010). Two new species of Myxobolus (Myxosporea, Bivalvulida) from the Indian major carp Labeo rohita Hamilton, 1822. Protistology.

[23] Kaur H, Singh R (2011). Two new species of Myxobolus (Myxozoa: Myxosporea: Bivalvulida) from freshwater fishes of Punjab Wetlands (India). J Parasit Dis.

[24] Kaur H, Singh R (2011). Two new species of Myxobolus (Myxozoa: Myxosporea: Bivalvulida) infecting an Indian major carp in Ropar and Kanjali wetlands (Punjab). J Parasit Dis.

[25] Kaur H, Singh R (2011). Myxobolus harikensis sp nov (Myxozoa: Myxobolidae) infecting fins of Cirrhina mrigala (Ham) - An Indian major carp in Harike Wetland, Punjab (India). Parasitol Res.

[26] Kaur H, Singh R (2011). Two new and one already known species of Myxobolus (Myxozoa:Myxosporea: Bivalvulida infecting gill lamellae of Indian major carp fishes in Ropar and Harike wetlands (Punjab). Proc 22nd Nat Cong Parasitology.

[27] Kaur H, Singh R (2011). Two new species of Myxobolus (Myxozoa: Myxosporea: Bivalvulida) infecting Indian freshwater fishes in Punjab Wetlands (India). Parasitol Res.

[28] Lom J, Arthur JR (1989). A guideline for the preparation of species descriptions in Myxosporea. J Fish Dis Oxford.

[29] Barta JR, Martin DS, Liberator PA, Dashkevicz M, Anderson JW, Feighner SD, Elbrecht A, Perkins-Barrow A, Jenkins MC, Danforth HD, Ruff MD, Profous-Juchelka H (1997). Phylogenetic relationships among eight Eimeria species infecting domestic fowl inferred using complete small subunit ribosomal DNA sequences. J Parasitol.

[30] Thompson JD, Gibson TJ, Plewniak F, Jeanmougin F, Higgins DG (1997). The ClustalX windows interface: flexible strategies for multiple sequence alignment aided by quality analysis tools. Nucleic Acids Res.

[31] Tamura K, Dudley J, Nei M, Kumar S (2007). MEGA4: Molecular Evolutionary Genetics Analysis (MEGA) software version 4.0. Mol Biol Evo.

[32] Kimura M (1980). A simple method for estimating evolutionary rate of base substitutions through comparative studies of nucleotide sequences. J Mol Evo.

[33] Saitou N, Nei M (1987). The neighbor-joining method: A new method for reconstructing phylogenetic trees. Mol Biol Evo.

[34] Felsenstein J (1985). Confidence limits on phylogenies: An approach using the bootstrap. Evolution.

[35] Lalitha-Kumari PS (1969). Studies on parasitic protozoa (Myxosporidia) of freshwater fishes of Andhra Pradesh, India. Riv Parassitol.

[36] Gupta S, Khera S (1988). Review of the genus Myxobolus Butschli, 1882. Res Bull (Sci) Panj Univ.

[37] Kundu TK (1985). Myxosoma indirae spn (Myxozoa: Myxosomatidae) from the head cartilage,scale and tail fin of Cirrhina mrigala. Acta Protozool.

[38] Sarkar NK (1986). On two new species of Myxobolus Butschli, 1882 (Myxozoa, Myxosporea) from the fresh-water fishes of West-Bengal, India. Acta Protozool.

[39] Seenappa D, Manohar L (1981). Five new species of Myxobolus (Myxosporea, Protozoa), parasitic in Cirrhina mrigala (Hamilton) and Labeo rohita (Hamilton), with a note on a new host record for Myxosporea curmucae Seenappa and Manohar. J Protozool.

[40] Hamilton-Buchanan F ( 1822). An Account of the Fishes of River Ganges and its Branches.

